# A Multiliteracies Approach to Transforming Written Cases Into Video Case Studies in Rehabilitation Education: A Design-Based Study

**DOI:** 10.1177/23821205261448452

**Published:** 2026-08-04

**Authors:** Skye Nandi Adams, Nancy Barber, Kelly-Ann Kater

**Affiliations:** 1 Department of Speech Pathology and Audiology, School of Human and Community Development, Faculty of Humanities, Ringgold: 37707 University of the Witwatersrand, Johannesburg, South Africa

**Keywords:** case-based learning, digital teaching, rehabilitation education, curriculum design, educational innovation

## Abstract

**Purpose:**

Case-based learning (CBL) is widely used in rehabilitation and continuing medical education to support the development of clinical reasoning and reflective practice. However, traditional written cases may inadequately capture the multimodal, relational, and contextual complexity of real-world clinical encounters. This paper describes a design-based process for transforming written case studies into video-based cases using a multiliteracies framework to enhance CBL in rehabilitation education, with relevance to continuing medical education.

**Methods:**

A design-based research approach guided the iterative development of three video case studies: a pediatric case, an adult recovery case, and a developed traumatic brain injury case. The process followed five design thinking phases: discovery, interpretation, ideation, experimentation, and evolution. Multiliteracies principles informed the integration of visual, auditory, linguistic, and relational modes of learning. All videos were co-created to ensure clinical accuracy, authenticity, and feasibility within a resource-constrained setting. An expert review was also conducted with the videos to explore the acceptability, feasibility, and appropriateness of the videos.

**Results:**

The design process resulted in three contextually grounded video case studies that supported learners’ engagement with clinical reasoning, patient-centered care, and multidisciplinary perspectives. The design-based approach facilitated interdisciplinary collaboration and refinement, showing a scalable and cost-conscious model for educational innovation. Experts reported high feasibility and acceptability alongside strong perceived appropriateness of the videos.

**Conclusion:**

Transforming written cases into video-based case studies using a multiliteracies-informed, design-based approach offers a practical and transferable strategy for enhancing case-based learning in rehabilitation and continuing medical education. This approach supports reflective, empathetic, and contextually responsive clinical reasoning and provides a replicable framework for educators seeking to integrate digital media into health professions education.

## Introduction

The rapid growth of technology and the use of multiliteracies within a digital era have highlighted the need to look at how we train students and the ways in which we utilize technology in our teaching.^1^ These suggestions are something that need to be considered in rehabilitation education and the use of digitally supported tools as part of the curriculum.^2^ Rehabilitation education includes the education of physiotherapists, occupational therapists, speech-language therapists (SLTs), audiologists, psychologists, and dietitians, who all play an essential role in improving patient outcomes, ensuring continuity of care, and enhancing the efficiency of healthcare systems.^3^Video-based case learning is increasingly relevant in continuing medical education, where clinicians require flexible, context-rich learning opportunities that support reflective practice and clinical decision-making across diverse settings.

Often in rehabilitation education, case-based learning is used as the pedagogical approach to ensure alignment of theory with practice.^3,4^ Case-based learning is defined as an approach that places students at the center of the learning process, exposing students to “patient cases” to connect theoretical knowledge with practical application.^5^ Through structured exploration of these cases, learners are encouraged to integrate concepts, solve problems, and develop the clinical reasoning skills needed for professional practice.^5-7^ Cases are generally based on a real patient and a narrative surrounding that patient is constructed to increase authenticity and thus enhance student satisfaction with the learning experience.^8^ Furthermore, in case-based learning, the lecturer’s role is that of facilitator and not the expert.^6^ Their role is to guide students, through questions, to understand the case, problem solve, provide an accurate diagnosis and formulate applicable treatment plans. This facilitation can support the integration of prior knowledge with new information that the students are learning.^6^ However, in order to move teaching and learning forward there is a need to utilize the possibilities offered by new digital technologies such as video-based case studies and interactive multimedia platforms.^9^ Digital technologies offer opportunities to integrate visual, auditory and contextual information that support deeper clinical reasoning.

Multiliteracies focus on the ways in which we integrate not only written or printed texts but also explore a variety of modes in teaching. This approach has been used recently in education.^1,8^ Multiliteracies approaches acknowledge that literacy and learning are no longer just about reading and writing but includes multiple modes such as visual, audio, gestural, and linguistic communication, all of which are particularly relevant in clinical education and diverse, multicultural contexts.^10,11^ Pedagogically, multiliteracies are framed around four interrelated knowledge processes: (1) experiencing, (2) conceptualizing, (3) analyzing, and (4) applying.^8^ Experiencing involves connecting new learning to real-world or personal experiences and grounding understanding in authentic contexts. Conceptualizing requires learners to identify and articulate key ideas or patterns, linking concrete experience to theoretical knowledge. Analyzing engages students in critically examining meaning, purpose, and perspective, while applying emphasizes the transfer of knowledge to new or complex situations, fostering creativity and adaptability.^8^

In rehabilitation education, multiliteracies provide a valuable framework for adapting case-based learning from written texts to video productions of the case and the move to the digitization of teaching and learning. This framework allows educators to design case-based learning so that students engage with authentic, multifaceted clinical scenarios that require the integration of knowledge, reasoning, and empathy. Case-based learning naturally aligns with these knowledge processes: students experience and interpret patient narratives, conceptualize clinical issues through theory, analyze communication and contextual factors, and apply their understanding to propose interventions.^3,4,6^ By combining these approaches, educators can design learning experiences that mirror the multimodal and relational nature of clinical communication, promoting deeper learning that bridges theory and practice.^9^

A multiliteracies approach recognizes that language and communication practices are culturally situated. As researchers and educators working in low- and middle-income countries (LMIC), it is crucial to use material that is contextually and culturally relevant. Decolonization in this context involves challenging dominant power and knowledge structures to transform curricula, teaching methods, assessment practices, and research design.^10^ In line with this, the development of our own teaching material aims to empower lecturers and clinical educators with resources that reflect local realities and cultural perspectives.^11^ As part of this research, we aim to explore equal access to education through the adaptation of the learning environment.

This research aims to enhance case-based learning in medical and rehabilitation education by transforming written case studies into engaging video-based learning materials using a multiliteracies approach.

## Methodology

### A Design-Based Approach

This paper used a design-based approach to describe the ways in which we incorporated a multiliteracy approach in the development of video case studies to be used in the teaching and training of SLT students. This project was in response to multiple challenges, including the need to bring about a pedagogic shift to meet the needs of a changing curricular through the integration of diverse forms of communication and meaning-making beyond traditional text-based learning.^12^ We chose video case studies as these combine visual, audio, and spoken language to represent real-life clinical scenarios.

**Figure 1.**
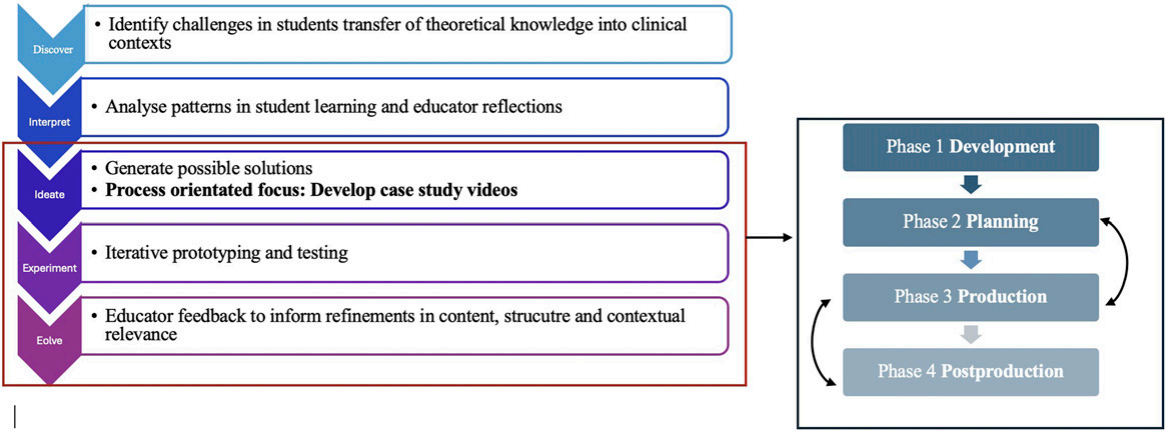
A design-based research process for developing video case studies in clinical education

The design-based approach enabled systematic documentation of educational decision-making, supporting transparency and transferability for educators designing continued professional development and rehabilitation training materials. It allows theory and practice to inform each other while addressing authentic challenges through contextually grounded interventions.^13^ To respond meaningfully to the challenges observed in our teaching context, we drew on principles of design thinking, which emphasize empathy, experimentation, and collaboration through five phases of the design thinking cycle^14^ (Figure 1). Drawing on the principles of design thinking, our process began with the *discovery phase*, where we identified challenges in students’ ability to apply theoretical knowledge to clinical practice through departmental discussions and insights from earlier work by Adams et al^12^ on developing a case-based learning framework in medical education. During the *interpret phase*, we explored patterns in student learning and educator feedback to clarify the underlying barriers to knowledge integration. In the *ideate phase*, we generated possible solutions and determined that converting written case-based materials to video case materials could potentially bridge the gap between classroom learning and clinical application. The *experiment phase* involved developing and testing prototypes of written case studies, which then evolved into video case studies to better capture the complexity of real clinical encounters. Finally, in the *evolve phase*, iterative feedback from educators in our research group informed ongoing refinement of these materials to ensure their pedagogical and contextual relevance.

Within this approach, the paper adopts a process-oriented focus, reflecting on the development of video case studies during the *ideate, experiment* and *evolve phases* as tools that integrate theory, practice, and communication within authentic learning contexts. The development during these phases followed two approaches. The first was developing a written case with clinical educators and turning that into a video, and the second was using a real patient and developing a video around their story. In total, we worked through five stages as outlined below to ensure *ideation*, *experimentation,* and *evolving* as aligned with a design-based approach.

The reporting of this study conforms to the Standards for QUality Improvement Reporting Excellence (SQUIRE 2.0)^15^ (Evidence of the guideline in supplementary document 1). The SQUIRE guidelines were used to structure the reporting of this educational intervention, as the study aimed to generate transferable knowledge on how educational innovations in allied healthcare professions education can strengthen clinical training and, in turn, support improvements in healthcare practice.

### Phase 1: Development

Ethics approval was granted by the University of the Witwatersrand Human Research Ethics Committee (approval number: HRECNMW24/06/03). The first phase was the development of a written case study. We developed a framework^16^ to guide the creation of the cases with 8 clinical educators. All participating clinical educators had a degree in SLP and had postgraduate qualifications, specifically 3 had a master’s degree and 5 had a PhD. Clinical educators had on average 19 years of experience ranging between 12 – 33 years. The focus of this session was to get input from clinical educators on what students were seeing at the clinics. The workshop was also used to explore the perception of clinical educators regarding challenges with clinical practice, theoretical gaps and input on the ways in which they would incorporate a video into their teaching.

A second part of the first phase was to identify appropriate patients that we could use to develop three case study videos. We wanted to use real patients to represent one paediatric case, one adult case and one dramatized case using actors. For the real patient cases, clinical educators and clinical sites were approached and asked if they had any patients that would be willing and able to share their story via film. Patients were chosen based on their representation of what students would be seeing at clinics and each video had a slightly different focus and outcomes so that we were able to provide students with a range of cases to support their learning and development. For the dramatized case, we consulted with the clinical educators to identify a case that is commonly seen at the student’s practical site and to highlight key aspects that they would like students to be able to understand, apply, analyse, evaluate, and create. These five criteria were chosen to align with Blooms taxonomy.

### Phase 2: Planning

The second stage was working with a production company to create the videos. Each video was co-created with the production company to ensure the videos were theoretically accurate but also visually appealing. This approach should allow students to engage with diverse communication and clinical reasoning challenges that reflected the realities of South African practice, including linguistic diversity, inequitable access to healthcare, and culturally grounded understandings of health and disability. By doing so, the videos should support the development of contextually responsive and empathetic clinical reasoning skills, preparing students to provide person-centred care within complex and diverse local contexts (Table 1).

**Table 1.** Videos and Outcomes

Video	Focus	Outcomes	People involved	Site	Filming time	Unique advantages of video case
Video 1	School-aged child with autism; includes perspectives from father and teacher across home–school environments.	• Appropriate assessment and management based on case information from multiple perspectives.	Child and his father and the class teacher	At the school	Half a day (morning)	Enables observation of the child’s communication behaviours within a real context, including how these behaviours present across everyday interactions. Highlights the father’s perspective and adjustment process, allowing learners to engage with how families make sense of the diagnosis and its impact. This supports understanding of the lived experience of autism in a way that is not accessible through written case descriptions.
	Background: Child is 4 years old and diagnosed with autism when he was 2. Presenting with limited verbal communication, restricted social interaction, and feeding selectivity.	• Appropriate assessment and management of communication and feeding difficulties.	​	​	​	​
	​	• Ability to show clinical reasoning and clinical integration.	​	​	​	​
Video 2	Adult post stroke recovery journey; perspectives from patient and mother. Reflects recovery trajectory, functional challenges, and home-based participation	• Integrated healthcare journey focusing on patient centred care.	Patient and her mum	At the patient’s home	Half a day (afternoon)	Highlights lived experience through narrative, emotion, and environment. Allows learners to see the impact of communication difficulties on identity and participation, and to engage with authentic storytelling, tone, and affect, which are difficult to convey through text alone.
	Background: Adult post-stroke patient with acquired communication difficulties. Experiencing changes in functional communication, identity, and daily participation.	• Understanding the person behind the diagnosis.	​	​	​	​
	​	• Ability to show clinical reasoning and clinical integration into assessment and management planning.	​	​	​	​
Video 3	Developed case. This video focused on a traumatic brain injury (TBI) case of a student and seeking support from the speech clinic at the university. Simulated clinical consultation	• Diagnosis, case history, counselling, and assessment planning for patient with TBI	Three student actors (the patient, the patient’s girlfriend, and the SLP)	At the university (staged therapy room)	Full day	Demonstrates temporal flow of a clinical interaction, including turn-taking, breakdowns, and repair. Allows learners to observe counselling strategies, behavioural responses, and clinician decision-making in action, supporting application of skills in a way that static written cases cannot replicate.
	Background: Young adult with TBI presenting cognitive-communication impairments, including impulsivity, reduced insight, and behavioural dysregulation.	• Long term outcomes and therapy management	​	​	​	​
	​	• Ability to show clinical reasoning and clinical integration.	​	​	​	​
	​	• Integrated healthcare journey focusing on patient centred care.	​	​	​	​

Across all three video cases, the primary educational outcomes targeted included clinical reasoning, patient-centred decision-making, and integration of multidisciplinary perspectives. The video format enabled learners to engage with temporal sequencing, communication dynamics, and contextual influences that are essential for professional practice but difficult to convey through written cases alone.

For the patient videos, the interview questions were purposefully designed to guide the development of authentic and contextually grounded cases. This process aligned with case-based learning principles, as it ensured that the resulting cases reflected real-world clinical and communicative challenges that students could critically engage with. In addition, by allowing participants to share their stories in their own words, the multiliteracies framework was reflected, as that framework values diverse modes of meaning-making which includes spoken narrative, emotion, and lived experience, as essential to understanding communication in context the process embodied the multiliteracies framework.^9^ Through this process, the videos became not only clinical teaching tools but also narrative representations of real-world complexity and human experience that are relevant to the students’ context.

For the developed case we used the information provided by the clinical educators and we created an initial script using ChatGPT. ChatGPT was an appropriate method to assist us with starting the script as we had no access or funding for script writers. ChatGPT was found to be particularly useful, and we recommend it for others who are working in contexts where educators do not have access or finances to a scriptwriter. ChatGPT was provided with an initial prompt of: “Write a script for a speech therapist doing an initial interview with a young adult woman after a TBI and her girlfriend. The woman is aggressive and impulsive”. ChatGPT was also provided with cues to make a script for assessing the patient with the Cognitive Linguistic Quick Test and a third script for collaborative goal setting. These three aspects of a session were created to provide students with insights into how a session can progress, aligning with case-based learning principles that emphasise learning through authentic, sequential problem-solving.^3^ We then reworked the script to ensure it was contextually and linguistically appropriate for our context.

To ensure that the AI-generated content aligned with clinical best practice, ethical standards, and educational objectives, a multi-step review and refinement process was undertaken by the research team. Scripts were reviewed by experienced clinicians to confirm clinical accuracy and alignment with evidence-based and competency-based frameworks. They were then adapted for contextual, cultural, and linguistic relevance to the South African setting and evaluated for ethical representation of patients. Final scripts were iteratively refined through collaboration between the research team, clinical educators, and production partners. Throughout this process, ChatGPT served as an initial drafting tool, while human expertise guided validation, contextualisation, and final decision-making.

The final script (supplementary material 2) was then used to create a brief of each of the three characters in the video. We partnered with the Department of Drama at the University of the Witwatersrand and drama students auditioned for the roles. Auditions were held with the researchers, the production company, and the coordinator of the final year drama students. Students were chosen based on an *Audition Checklist: Student Actor for Training Video* (supplementary material 3). The audition checklist had 8 criteria that the students were assessed on and included (1) Appearance & screen presence, (2) Voice & diction, (3) Performance quality, (4) Reading & memorisation, (5) Comprehension and interpretation, (6) Professionalism & attitude, (7) Professional fit, and (8) Availability & practical considerations. The students were given two weeks to learn their lines and a run through was scheduled with the researchers, student actors and production company a week before filming. In addition, locations were scouted before filming to ascertain where filming could take place, and all permissions were received prior to filming from the different sites and participants.

### Phase 3: Production

Filming took place over two days. The real cases were videoed over one day. The paediatric case was filmed in the morning to accommodate the school times, and the adult case was filmed in the second half of the day at the patient’s home. Videos with patients were cocreated to allow patients and caregivers to share their story. This approach aligns with case-based learning by grounding teaching materials in authentic, lived experiences that students can analyse, reflect on, and apply to future clinical encounters.^3^ From a multiliteracies perspective, storytelling represents a rich mode of meaning-making that extends beyond verbal content to include emotion, tone, and context.

The second day of filming was set aside for the developed case, and it was filmed on campus in the therapy rooms. This developed case aligned with a case-based approach as it aimed to simulate an authentic clinical environment, enabling students to engage with realistic scenarios that mirror professional practice. The developed case also reflected the principles of multiliteracies by combining verbal, visual, and embodied forms of communication to construct meaning. The director from the production company provided students with detailed feedback on their performances, supporting iterative learning and refinement of the video.

### Phase 4: Postproduction

**Figure 2.**
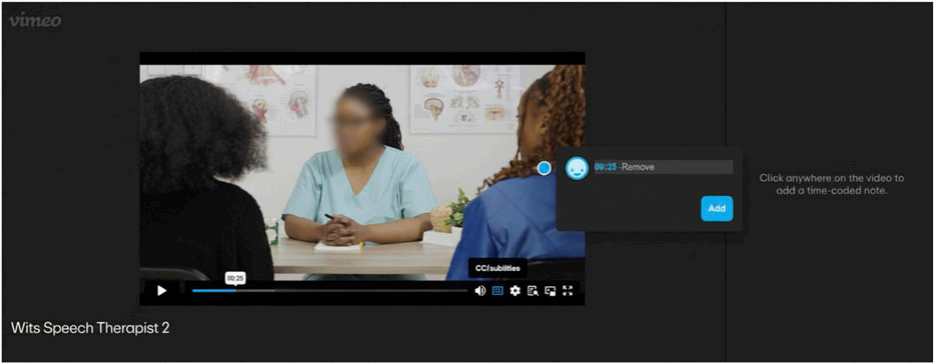
Example of video stringouts and researcher comments

Once filming was completed the production company sent through video string outs of each interview. The research team decided on which string outs to include in the final video which would be 5 minutes long. Feedback was sent to the production company (Figure 2) which allowed the researchers to comment on any changes they would like. The production company then created the video including the final string outs and additional footage that was filmed to provide context for the case being presented by the video.

As part of the review and refinement process, the video case studies were evaluated by five subject matter experts who were lecturers and clinical educators within the Speech-Language Pathology department and actively involved in both academic teaching and clinical training. Experts were selected based on the following criteria: (1) a minimum of three years’ experience in clinical teaching and supervision (2) active involvement in academic and clinical training, and (3) experience using case-based learning in rehabilitation education. All reviewers were familiar with competency-based clinical training requirements. The expert review was guided by the following research question: How acceptable, feasible, and appropriate are the video-based case studies for use in case-based learning within rehabilitation and continuing medical education, as perceived by clinical educators and academic staff?

Feedback was analysed using the Acceptability of Intervention Measure (AIM), Feasibility of Intervention Measure (FIM), and Intervention Appropriateness Measure (IAM) frameworks^13^ to systematically examine educators’ perceptions of the video case studies as an educational intervention. The AIM, IAM and FIM are each four-item measures, which can be administered to healthcare professionals to determine the extent they believe a resource is acceptable, appropriate and feasible. Cut-off scores are not yet available for the AIM, IAM and FIM. However, higher scores indicate greater acceptability, appropriateness and feasibility. Experts were also asked to provide feedback on their general thoughts of the video, what they would change and how they feel they might be able to use it in either theoretical or clinical teaching.

## Results

The design-based process resulted in the development of three video-based case studies for use in rehabilitation education and continuing medical education. Each case was designed to support case-based learning by enabling learners to engage with clinical reasoning, patient-centred decision-making, and contextualised communication across diverse rehabilitation contexts. Across all cases, the videos were structured to foreground clinical decision points, multidisciplinary perspectives, and sequential reasoning processes relevant to assessment, diagnosis, and management. Each final video was approximately five minutes in duration, ensuring feasibility for integration into classroom-based, blended, or continuing education settings. It is important to note that due to the small number of subject matter experts involved, the evaluation findings should be interpreted as preliminary and exploratory, providing initial insights into the acceptability, appropriateness, and feasibility of the video case studies rather than definitive conclusions.

### Expert Review Process

**Figure 3.**
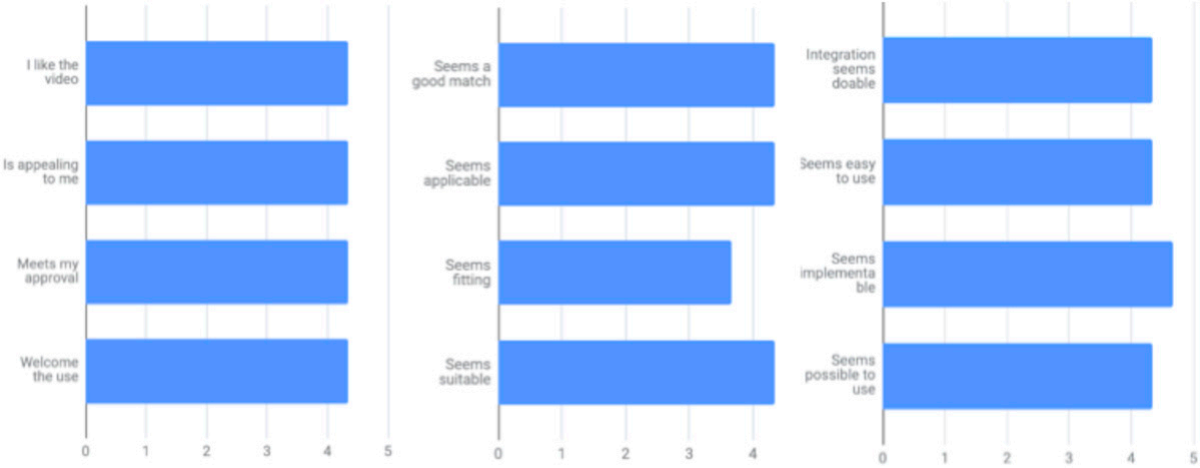
Mean scores for individual items of the Acceptability of Intervention Measure (AIM), Intervention Appropriateness Measure (IAM), and Feasibility of Intervention Measure (FIM) (n = 5). Scores were rated on a 5-point Likert scale (1 = completely disagree; 5 = completely agree). Higher scores indicate greater perceived acceptability, appropriateness, and feasibility

The following results reflect clinical educators’ and academic staff’s evaluations of the video case studies using the AIM, IAM, and FIM frameworks (Figure 3).

### Acceptability of the Video Case Studies (AIM)

The average score for AIM was 4.2/5 indicating that the videos were highly acceptability, with some variability reflecting differing expectations of clinical detail. Most participants strongly agreed that the video met their approval, was appealing, and was a welcome addition to teaching resources. Lower scores were primarily associated with reviewers expressing a desire for additional observations of communication behaviours in the videos.

### Intervention Appropriateness Measure (IAM)

The average score for IAM was 4.3/5. The video was broadly perceived as appropriate for its educational purpose. Reviewers identified the case as suitable and applicable for teaching clinical reasoning, contextual responsiveness, and multidisciplinary engagement, particularly within culturally diverse settings.

### Feasibility of Intervention Measure (FIM)

The average score for FIM was 4.4/5. Feasibility was rated highest across the three domains, indicating strong perceived practicality for integration into teaching and training contexts. Reviewers indicated that the short duration and flexible format supported use in lectures, case discussions, reflective activities, and assessment contexts.

Quantitative analysis of educator feedback using the AIM, IAM and FIM demonstrated overall positive perceptions of the three videos. Open-ended responses reinforced quantitative findings. Acceptability was supported by comments describing the video as “engaging,” “excellent,” and “a culturally and contextually valuable teaching tool.” Suggestions for improvement focused on expanding visual representation of the child’s communicative behaviours rather than concerns about the video format itself. Appropriateness was reflected in reviewers’ descriptions of the video as valuable for preparing students to work with autistic children and for supporting summative assessment and reflective discussion. Feasibility was further supported by reviewers stated intentions to use the video in lectures, case history teaching, and clinical training activities.

## Discussion

The design process demonstrated that video-based case studies could be developed within a resource-constrained educational context through interdisciplinary collaboration and phased design. Innovation in technology and the increasing emphasis on multiliteracies within higher education have transformed how educators conceptualise and deliver learning experiences.^1,12^ In this study, a multiliteracies-informed, design-based approach was used to develop video case studies intended to complement existing case-based learning approaches in rehabilitation education. By integrating video case studies grounded in real South African contexts, we sought to reimagine case-based learning as a multimodal, socially responsive, and pedagogically transformative process.^17^

The findings suggest that video case studies may extend traditional case-based learning by incorporating multiple modes of information, including visual, auditory, and contextual cues. In traditional case-based learning, students read written cases, analyse them, and apply theory to practice.^3,6^ The multiliteracies framework extends this by encouraging learners to interpret meaning across visual, oral, gestural, and spatial modes.^9,18^ Video cases, therefore, allow students not only to *read* a case but also to *see* and *hear* the lived experiences of patients, caregivers, and clinicians.^18,19^ Through this multimodal engagement, students can observe communicative nuances such as tone, body language, and emotional expression which are key competencies in rehabilitation practice.^20,21^ Beyond pedagogical enrichment, the use of multiliteracies supports the broader goals of transformation and decolonisation in South African higher education. Locally produced video cases represent patients and caregivers from linguistically and culturally diverse communities, addressing long-standing gaps in diversity and representation.^10,11^ These narratives challenge Western-centric epistemologies that have historically shaped medical curricula and create opportunities for students to engage with the social and structural determinants of health that shape clinical encounters in South Africa.^22,23^

The design-based process used to develop the video cases further exemplified how innovation can emerge through iterative, collaborative, and reflective practice.^13,14^ It allowed us to move systematically from identifying pedagogical challenges to creating, testing, and refining solutions within authentic educational contexts.^13^ Importantly, the iterative feedback loops built into the design process supported adaptive thinking, allowing the team to refine materials in response to stakeholder input, contextual limitations, and evolving pedagogical insights. The design-based process also exemplified how innovation can emerge through cross-disciplinary and collaborative engagement. Working with clinical educators, drama students, and a production team reflected the collaborative nature of both design-based and multiliteracies pedagogies, which value diverse forms of expertise and meaning-making.

Co-creating videos with patients and caregivers added a powerful dimension of storytelling. Storytelling, as a mode of multiliteracy, that situates learning in a lived experience.^1,24^ These video case studies highlight how educational processes can also become spaces of human connection and for students, engaging with such narratives, it humanises the clinical process which in turn should foster reflective awareness.^25^

While the production of video case studies requires resources, this project demonstrated that meaningful collaboration could mitigate costs and enhance cross-disciplinary learning. Partnering with departments such as the Department of Drama allowed for shared expertise in script development, performance, and production. This approach not only built capacity within the institution but also offered students in different disciplines opportunities for experiential learning and creative problem-solving which are values central to both design thinking and multiliteracies pedagogy.^12,14^ Furthermore, leveraging artificial intelligence for initial script development proved valuable in resource-limited settings, supporting educators to generate adaptable, contextually appropriate materials.

The combined use of AIM, IAM, and FIM enabled structured educational evaluation by shifting the focus from effectiveness to implementation readiness, which is particularly appropriate at early stages of educational innovation.^9,12^ By applying these frameworks, the study contributes to a growing body of health professions education literature that advocates for the use of implementation science tools to guide the design, refinement, and adoption of educational interventions. Importantly, high feasibility and acceptability alongside strong perceived appropriateness suggest that video-based case studies represent a promising strategy for enhancing case-based learning in rehabilitation education. In the current study there was found to be a use for the videos both with training students and clinical educators. These findings reflect implementation readiness rather than effectiveness and support the potential use of video cases within both undergraduate training and continuing professional education.

Overall, this study contributes to the growing literature on digital and multimodal approaches to case-based learning in health professions education. The findings suggest that a design-based, multiliteracies-informed approach can support the development of contextually relevant and practically feasible educational resources. Further research is required to evaluate the impact of such resources on learner outcomes and their sustained use in educational practice.

### Limitations and Future Directions

Several limitations should be considered when interpreting the findings of this study, which also inform key priorities for future research. Firstly, the study adopted a design-based research approach focused on the development and refinement of educational materials rather than on evaluating educational effectiveness. While this approach is well suited to early-stage educational innovation and aligns with the study’s aims, it does not allow for conclusions about the impact of the video case studies on learner outcomes, clinical reasoning performance, or professional competence. A key priority for future research is to evaluate educational effectiveness, including learner-facing studies and comparative designs examining video-based versus written case formats.

Secondly, the evaluation was based on feedback from a small sample of subject matter experts (n = 5) within a single academic department. Although appropriate for preliminary implementation assessment using AIM, FIM, and IAM, the findings remain exploratory and not generalisable. Future studies should explore the impact of these video cases on student learning outcomes and their potential for broader implementation across health professions education and include feedback from different stakeholders involved including students, to get a more comprehensive and objective understanding of current reality.

Lastly, the study focused primarily on educator perceptions of acceptability, feasibility, and appropriateness, rather than on actual implementation or sustained use in educational practice. As such, the findings reflect implementation readiness rather than real-world adoption. A further priority is to examine how video case studies are integrated into curricula over time, including fidelity of use, adaptability, and sustainability in both undergraduate and continuing medical education contexts.

### Recommendations

Educators in rehabilitation and continuing medical education are encouraged to integrate video-based case studies as a complement to traditional written cases, particularly when teaching clinical reasoning, communication, and patient-centred care. Short, focused video cases appear especially suitable for lectures, tutorials, reflective discussions, and case-based assessments, where multimodal engagement can enhance understanding of real-world clinical complexity. Where feasible, educators should incorporate multiple perspectives within video cases, including those of patients, caregivers, and professionals, to support relational and contextual learning. When direct patient footage is limited, carefully designed simulated cases can be used to illustrate specific competencies and decision-making processes.

### Conclusion

This paper demonstrated how combining a multiliteracies approaches with case-based learning can support the creation of contextually relevant video case studies for rehabilitation education. By grounding design and pedagogy in the realities of South African healthcare and education, the project fostered authentic, multimodal, and inclusive learning experiences that bridge theory and practice. The process model developed here using a design-based approach, offers a replicable framework for other disciplines seeking to integrate technology, collaboration, and social responsiveness in curriculum design and continuing medical education.

## Supplemental Material

Supplemental Material - A Multiliteracies Approach to Transforming Written Cases Into Video Case Studies in Rehabilitation Education: A Design-Based Study Supplemental Material for A Multiliteracies Approach to Transforming Written Cases Into Video Case Studies in Rehabilitation Education: A Design-Based Study by Skye Nandi Adams, Nancy Barber and Kelly Ann Kater in Journal of Medical Education and Curricular Development.

Supplemental Material - A Multiliteracies Approach to Transforming Written Cases Into Video Case Studies in Rehabilitation Education: A Design-Based Study Supplemental Material for A Multiliteracies Approach to Transforming Written Cases Into Video Case Studies in Rehabilitation Education: A Design-Based Study by Skye Nandi Adams, Nancy Barber and Kelly Ann Kater in Journal of Medical Education and Curricular Development.

Supplemental Material - A Multiliteracies Approach to Transforming Written Cases Into Video Case Studies in Rehabilitation Education: A Design-Based Study Supplemental Material for A Multiliteracies Approach to Transforming Written Cases Into Video Case Studies in Rehabilitation Education: A Design-Based Study by Skye Nandi Adams, Nancy Barber and Kelly Ann Kater in Journal of Medical Education and Curricular Development.

## Data Availability

The data that support the findings of this study are available on request from the corresponding author. The data are not publicly available due to privacy or ethical restrictions.*
