# Supplementary material for: A Multiliteracies Approach to Transforming Written Cases Into Video Case Studies in Rehabilitation Education: A Design-Based Study
Source: J Med Educ Curric Dev. 2026 Aug 4;13:23821205261448452. doi: 10.1177/23821205261448452 (PMC13438337; doi:10.1177/23821205261448452)
Supplement: Supplementary file 2 [file sj-pdf-2-mde-10.1177_23821205261448452.pdf]

Supplementary material 2. Dramatized brief and script

Initial Interview Script: Speech Therapist, Young Adult Male (Impulsive/Aggressive) + Girlfriend

Brief: (mannerisms, appearance, dress code)

Actor 1 (Speech therapist-female)

Role Overview:

You play a Speech and Language Therapist (SLT), a trained healthcare professional who assesses, diagnoses, and supports individuals with communication and/or swallowing difficulties. Your role involves both clinical expertise and strong interpersonal skills. You work in an outpatient clinic, supporting a patient with traumatic brain injury (TBI).

Background and Professional Demeanor:

- Qualifications: Degree-level education in speech and language therapy; registered with a professional body (e.g., HPCSA).
- Personality: Empathetic, patient, attentive, and methodical. You're an excellent communicator, not just in speaking but in listening and observing non-verbal cues.
- Style: Calm and warm, but professional. You build trust with clients over time. You're analytical and work in structured ways but flexible enough to adapt your approach to individual needs.
- Approach: Holistic, patient-centered, and evidence-based. Therapy sessions are goal-oriented but highly adaptable, often requiring creativity and emotional intelligence.

Performance Notes:

- Use gentle body language—soft eye contact, nodding, minimal sudden movements.
- Speak clearly and at a measured pace; model effective communication.
- Show professionalism even when dealing with emotion-heavy scenes.
- You're not "fixing" people—you're supporting them toward greater confidence and independence.

Setting and Session Style:

- Environment: A quiet outpatient therapy room. Clinical but comfortable. Minimal distractions.
- Session Dynamics: Session with patient and girlfriend. Session will involve case history interview and assessment task. Girlfriend is calm, supportive but nervous. Patient is aggressive, agitated and inappropriate.

Character Traits and Performance Notes:

- Tone: Calm, encouraging, and grounded. You're patient, even when the patient becomes frustrated

- Language: Clear, measured speech. Use simple, supportive phrasing when needed.
- Body Language: Warm, open posture. Maintain steady eye contact, use gentle gestures. Avoid rushed or abrupt movements.
- Emotional Intelligence: TBI recovery can be deeply emotional—expect moments where the patient may become angry, withdrawn, or overwhelmed. Your character remains a steady presence, balancing empathy with therapeutic goals.

Dress code:

- Scrub top (to be provided by us)
- Black pants
- Closed shoes
- Minimal jewelery

Optional Research Tips:

- Watch SLT therapy videos focused on TBI (e.g., YouTube channels like "The Brain Injury Association" OR https://www.youtube.com/results?search_query=traumatic+brain+injury+adult+patient OR https://www.youtube.com/results?search_query=traumatic+brain+injury+adult+patient+with+speech+therapist).
- Read or listen to interviews with SLTs working in neurorehab.
- Understand common challenges for TBI patients: attention deficits, slowed processing, social communication changes, and emotional volatility.

Actor 2 (Patient-male)

Character Profile:

- Name: Sipho (or name of choice)
- Age: Early 20s
- Background: A university student who sustained a traumatic brain injury (TBI) in a recent accident (car crash) Prior to the injury, he was bright, articulate, and socially active studying engineering. His speech, memory, and processing abilities have been affected. Takes time to find a word and uses "um" a lot and becomes very agitated.

Character Traits & Behavior:

- Agitated & defensive: Interrupts, scoffs, rolls his eyes.
- In denial: Makes dismissive comments about the therapy ("This is pointless" or "I don't need this").
- Impatient with himself: When struggling to speak or recall, he may become visibly frustrated—grimacing, clenched fists, or possibly hitting his thigh/table.
- Emotionally volatile: Anger may mask deep embarrassment, grief, or fear. flips between anger and sarcasm

- Conflicted: Deep down, he wants to improve, but his pride and trauma get in the way.

Physical & Emotional Notes:

- May fidget, rock slightly, or pace—uncomfortable in the clinical environment.
- Body language is closed off (crossed arms, avoiding eye contact) but might soften briefly if the therapist shows empathy.
- Aggressive posture – quick to lash out verbally, especially when he feels misunderstood, pitied, or pushed.

Key Relationship:

- Girlfriend (Emily or name of choice):
  She's been supportive but is emotionally exhausted. She's trying to stay positive and encouraging in the session, but Sipho resents her involvement. He snaps at her—even when she's trying to help.
- Important dynamic: Her presence heightens his frustration—he's ashamed of her seeing him like this. There's love there, but also tension and miscommunication.

Tone & Delivery:

- Sipho's tone should be irritated, quick to anger, and often sarcastic, but the subtext is fear and loss of control.

Dress code:

- Dress as typical student

Actor 3 (Girlfriend)

Character Profile:

- Character Name: Emily [can be changed]
- Age Range: Early 20s
- Background: She has been with her partner since before his accident. They share a deep emotional bond. She is his emotional anchor.
- Occupation: Full-time university student (engineering- in the same class as boyfriend/patient)

Scene Context:

- This is the first time her boyfriend is attending speech therapy since suffering a traumatic brain injury. His recovery has been turbulent. She's here to support him, but she's deeply anxious. He has recently shown episodes of verbal aggression, irritability, and mood swings, which she tries to understand as symptoms of his injury — but it's taking a toll.
- She's young, in uncharted emotional territory, trying to balance studies, caregiving, and an uncertain future with someone she still loves but hardly recognizes at times.

Emotional Landscape

- Worried and Guarded: She's walking on eggshells, unsure of what mood he'll be in, afraid he might lash out — especially in unfamiliar settings like therapy.
- Exhausted and Conflicted: She loves him, but doubts are creeping in. Is she equipped for this? Is it fair to her?
- Protective but Wary: She wants to advocate for him, but also set boundaries for her emotional safety.
- Guilt-Ridden: She sometimes wishes it had never happened — and then feels ashamed for thinking that.
- Mature Beyond Her Years: She's been forced to grow up quickly, often hiding how overwhelmed she truly is.

Physicality & Behavior

- Often alert to his mood shifts; watches him closely for signs of escalation.
- Speaks gently but cautiously, always gauging his response.
- May flinch slightly when he raises his voice, even if not directed at her.
- Keeps a supportive posture, but her body is slightly tense — she's never fully relaxed.

Tone of the Scene

- A quiet storm of tension, love, and quiet dread. The room is clinical, but her emotional atmosphere is delicate and loaded.

Dress code:

- Dress as typical student
- Carry a school bag

Setting: EC Therapy room on campus in the speech therapy department. The therapist sits across from the patient and his girlfriend. A third chair is available for her support, but she is not the primary respondent unless asked.

<u>SCRIPT</u>

Speech Therapist (ST):

smiling gently

Hi Sipho, my name is Thando and I will be your speech therapist. It is lovely to meet you. Before we begin, I just wanted to find out if it is fine if your girlfriend joins us today?

Patient:

shrugs aggressively

I don't care. Just get on with it.

ST:
Thanks, Sipho. So are you happy for her to be here and we can start getting some information about why you are here today?

Patient:
I said it doesn't matter, okay. Can you not understand me or something. *leans back, arms crossed*

ST:
turns to girlfriend
Okay, so are you okay with that? Please can you tell me your name.

Girlfriend:
Thanks, my name is Thandiwe but you can just call me Thandi.

ST:
Thank you Thandi. I know it's not always easy to come into a space like this, and I appreciate both of you being here.My role is to support communication and thinking skills that may have changed, whether that's due to injury, illness, or something else. Today I'll ask a few questions so I can better understand what's been going on.

pauses briefly to check engagement

Let's start simple. Can you tell me what brought you here?

Patient:
Because she made me. *gestures to girlfriend*
Says I don't listen. Says I talk over everyone and "can't control myself."

Girlfriend:
quietly
It's been really hectic since the accident.

ST:
nods calmly
Okay, thanks for sharing that. I know that can be tough to talk about.
Can you tell me a little about the accident? What happened?

Patient:
tense

Car crash. Not my fault. Some taxi pulled out in front of me. I walked away. They said I hit my head. Whatever.

ST:
Do you remember going to the hospital?

Patient:
gruff
Yeah. There were a few days. They scanned my head. Said I had a concussion or something like that, something worse than a concussion. Can't really remember I was really out of it.

ST:
Thanks – that helps. Since then, have you noticed any changes in how you speak, think, or interact with people?

Patient:
interrupts
People say I've changed. I say what I want. I don't take crap anymore.

ST:
Okay. Has anything been harder – like following conversations, remembering things, or finding the right words?

Patient:
Maybe. People mumble. I lose track of stuff sometimes and its hard to sometimes follow what people are saying. But that's normal.

ST:
That's useful to know.
to girlfriend, gently
Would it be okay if I ask for your perspective as well? Only if you're comfortable.

Girlfriend:
Sure. He gets frustrated quickly. He'll start yelling over small things. He's not the same – his patience is gone. But I know it's not his fault. In class he is fidgety and very distracted. He cant sit still. He used to do so well in tests and assignments and now his grades are dropping I am really worried because he is on a bursary. He says he doesnt need to tell his mother about his grades and he that he will study harder.

Patient:

snaps

I'm right here, you know. Stop talking like I'm not.

ST:

calmly, redirecting

You're absolutely right. Let's keep the focus on what you are going through. .

If I ask her things, it's just to help me build the full picture. But your voice is the most important one here.

pauses to de-escalate

Let's talk about your day-to-day. Are you working, studying, or doing anything structured?

Patient:

Lecturers have said I am "disruptive." but I am just getting annoyed with the lecturers always telling me what to do and...and...ya.. Its annoying when I cant do everything. I am not stupid but they make me feel like I am.

ST:

Understood. It's common after a head injury for learning or social situations to feel more difficult. What do you want to be able to do again, or do differently?

Patient:

pauses

I just want people to stop treating me like I'm crazy. I'm not. I'm just... tired of feeling like everything is a fight.

ST:

That's really important to hear. You're not alone in feeling that way. What we can do together is look at where you feel stuck, and build strategies that work for you.

Would you be open to that?

Patient:

sighs, softer tone

I guess. Depends what you make me do.

ST:

Fair enough. We'll work at your pace.

For today, I've just got a few more short questions so I can put together a plan. Is that okay?

Patient:
Yeah. Just don’t waste my time.

ST:
I hear you. I’ll keep it focused.
Let’s start with…

Snippet of Assessment

- Assessment – QCLT
- Story retelling

CLQT Subtest 5: Story Retelling (Script with Responses)

Setting: Quiet therapy room. Materials ready. Therapist seated across from Sipho. Tone is calm and supportive.

ST:
Okay, for this next task, I’m going to read you a short story. You only need to listen right now — don’t interrupt or take notes. When I’m done, I’ll ask you to tell me everything you remember. I cannot repeat the story so try to remember as much as possible.

Are you ready?

Patient:
sighs, leaning back
Yeah, just read it.

ST:
Reads clearly and evenly

"Mrs Brown lives in a small white house on the corner of Oak Street. Every morning, she walks her dog to the park and sits on the same green bench. One morning, the bench was missing. She looked around, confused. Later, she found out the bench had been removed by the city for repairs."

ST:
Pauses for a moment
Alright. Please tell me everything you remember from that story.

Patient:

speaks quickly

There was a woman… she had a house. And… something about a dog? And a bench?

ST:

Okay great. Can you tell me anything else?

Patient:

frustrated

Uh… I don't know. You just said it, surely you know that story! I think something also happened to the bench. It was gone or whatever.

ST:

Yes that's part of it. Now can you remember what was her name?

Patient:

shrugs

Um... Um.. No idea. Does it really matter? I am bad with names anyway

ST:

That's okay. Do you remember which street she lived on?

Patient:

pauses

Um... Oak? Oak something?

ST:

Yes — Oak Street. And where did she go with her dog?

Patient:

The park. Every day, right?

ST:

That's it and what did she usually do there?

Patient:

Um She sat on a bench um. The same one every time. But now it was gone.

ST:

And do you remember what colour the bench was?

Patient:

thinking hard

Green?

ST:

Yip. And why was the bench gone?

Patient:

Uh… the city took it. To fix it?

ST:

Yes — for repairs. You remembered quite a bit from that story. Let's go on to our final task

Collaborative goal setting:

Speech Therapist (ST):

Okay, you've done a lot of work today, and I appreciate you trying. I wanted to spend a few minutes talking with you about something you mentioned earlier — getting back to your studies.

Patient:

tone uncertain or defensive

Yeah, maybe. I don't know if I'm ready. It's hard to concentrate now.

ST:

That makes total sense. A brain injury can affect things like memory, focus, and organisation — all of which you need when studying. But we can work on that together. Would you be willing to set some goals so that you can go back to study again when you're ready?

Patient:

sighs

Maybe. It depends what you mean by "goals."

ST:

Fair enough. I'm not talking about huge **goals**. We have to start with small, realistic steps that we come up with together that eventually lead to the end goal of being able to study again. We have to choose things together that feel doable for you and help you get closer to studying, one step at a time.

what are you studying?

Patient:

I am in my 2nd year of engineering. . I was halfway through.

ST:

What kind of skills do you need to be able to study again? When I was giving you feedback about the assessment I noted that you had some difficulty concentrating and you agreed with me. Is that a skill you need to return to studying?

Patient:

Definitely. I just want to be able to focus long enough to finish my work without freaking out or getting stuck.

ST:

That's a great start — being able to focus, feel in control, and finish tasks. We can build some short-term goals based on that. Here's a way we can do it:

Let's make each goal:

Specific – so you know exactly what you're working on

Measurable – so we can see progress

Achievable – so it's something you can actually do

Relevant – so it fits your real-life needs

Time-bound – with a realistic timeline

Let's try one together.

[End]
