# Supplementary material for: A Multiliteracies Approach to Transforming Written Cases Into Video Case Studies in Rehabilitation Education: A Design-Based Study
Source: J Med Educ Curric Dev. 2026 Aug 4;13:23821205261448452. doi: 10.1177/23821205261448452 (PMC13438337; doi:10.1177/23821205261448452)
Supplement: Supplementary file 3 [file sj-pdf-3-mde-10.1177_23821205261448452.pdf]

Supplementary material 3. Audition Checklist: Student Actor for Training Video

1. Appearance & Screen Presence

- Appropriate look for the role (age, demeanour, relatability)
- Natural on camera — not overly self-conscious
- Clear and expressive facial expressions

2. Voice & Diction

- Clear articulation and pronunciation
- Good projection (audible without shouting)
- Natural-sounding delivery (avoiding a “stagey” or flat tone)

3. Performance Quality

- Believability and authenticity in delivery
- Ability to take direction and adjust performance
- Confidence in role play or scenario-based acting
- Emotional control and consistency

4. Reading & Memorisation

- Ability to read script smoothly (if reading on camera)
- Retains lines or content well
- Doesn’t rely too heavily on cues
- Comfortable with scripted dialogue

5. Comprehension & Interpretation

- Demonstrates understanding of the script tone and context
- Can portray the character convincingly

6. Professionalism & Attitude

- Punctual and prepared
- Receptive to feedback
- Respectful, focused, and engaged
- Demonstrates enthusiasm and willingness to learn

7. Technical Fit

- Voice and delivery work well with mic and camera setup
- Physically comfortable in front of camera/lighting set up

8. Availability & Practical Considerations

- Available for shoot dates and any necessary rehearsals
- Comfortable with filming duration and setup
- Willing to sign release form or appearance agreement
